# Black Women’s Perceptions Towards Infant and Child Male Circumcision

**DOI:** 10.1007/s10995-023-03693-6

**Published:** 2023-06-05

**Authors:** Eurica Palmer, Lochner Marais, Michelle Engelbrecht

**Affiliations:** 1 Centre for Development Support, Faculty of Economic and Management Sciences, University of the Free State, Bloemfontein, South Africa; 2 Centre for Health Systems Research & Development, University of the Free State, Bloemfontein, South Africa

**Keywords:** Medical male circumcision, Social Norms Theory, Traditional male circumcision

## Abstract

**Objective:**

The objective of this article was to analyse women’s perceptions of ICMC and to propose a framework for ICMC decision-making that can inform ICMC policies.

**Method:**

Using qualitative interviews, this study investigated twenty-five Black women’s perceptions of ICMC decisionmaking in South Africa. Black women who had opted not to circumcise their sons, were selected through purposive and snowball sampling. Underpinned by the Social Norms Theory, their responses were analysed through in-depth interviews and a framework analysis. We conducted the study in the townships of Diepsloot and Diepkloof, Gauteng, South Africa.

**Results:**

Three major themes emerged: medical mistrust, inaccurate knowledge leading to myths and misconceptions, and cultural practices related to traditional male circumcision. Building Black women’s trust in the public health system is important for ICMC decision-making.

**Conclusions for Practice:**

Policies should address misinformation through platforms that Black women share. There should be an acknowledgement of the role that cultural differences play in the decision-making process. This study developed an ICMC perception framework to inform policy.

## Significance

*What is already known on this subject*? Male circumcision decision-making is complex because multiple, interrelated factors influence it. Deciding about circumcising infants and children is a challenge that many parents face.

* What this study adds*? This study found that social pressure from the social network, myths and misconceptions resulting from inaccurate knowledge, medical mistrust and traditional male circumcision practices influence the decisions of Black women not to circumcise their sons. The study proposes a perceptions framework to demonstrate consideration for Black women’s voices in the male circumcision discourse.

## Introduction

Globally, male circumcision has gained momentum as a strategy for preventing HIV transmission. Randomised controlled trials found that adult medical male circumcision reduces the risk of HIV infection in heterosexual males by approximately 60% (Auvert et al., 2005; Bailey et al., 2007). Several African countries have rolled out infant and child male circumcision (ICMC) programmes (Jarrett et al., 2014; Keetile & Bowelo, 2016). South Africa implemented its medical male circumcision programme in 2010, although the government only published the *South African National Guidelines for Medical Male Circumcision* in 2016 (South Africa & Department of Health, 2016). In contrast to South Africa, countries like Botswana and Eswatini have implemented ICMC programmes since 2014 and 2009, respectively (National Center for HIV/AIDS, Viral Hepatitis, STD, and TB Prevention, 2014; Swaziland, Ministry of Health & World Health Organization, 2014). However, ICMC as an HIV-prevention strategy requires acceptance by caregivers, including female caregivers. As a result, questions were raised among families of infants and children about how caregivers can make decisions about ICMC, as it is not yet known how female caregivers think or how they make decisions in this regard (Jarrett et al., 2014; Keetile & Bowelo, 2016). With 37.9% of all South African households headed by females, women’s involvement in ICMC decision-making is important (Statsistics South Africa, 2019).

The role of women in circumcision decision-making was emphasised in a joint strategic action framework by the Joint United Nations Programme for HIV/AIDS and the World Health Organisation (UNAIDS/WHO, 2011). However, in Eastern and Southern Africa, to increase the uptake of medical male circumcision, the target has been men. Governments ignored the advice of the UNAIDS/WHO about the role of women in providing support for men and adolescent boys in deciding about medical male circumcision (Nxumalo & Mchunu, 2019; Semeere et al., 2016). Perspectives on male circumcision programmes are narrowly focused on men (Segalo, 2015). The views of Black women seldom feature in policy documents. The lack of representation probably results from the multiple subordinate identities of Black women (Serrant, 2020). Therefore, male circumcision policies should consider the voices of Black women (Coles & Pasek, 2020).

The research on the role of women in the decisions about ICMC points to the influence of multiple factors, such as the father’s circumcision status, health and hygiene, the cost of the procedure and religious practices (Justman et al., 2013; Mavhu et al., 2014; Spyrelis et al., 2013). In a study in Eswatini, women described their consultation with their fathers as an important step in decision-making (Jarrett et al., 2014). The father’s circumcision status was also a positive decision-making factor among women in Botswana (Keetile & Bowelo, 2016). Moreover, South African and Zimbabwean studies found that masculinity and traditional male circumcision practices influenced women to reject ICMC (Mavhu et al., 2014; Rech et al., 2014). Social networks (peers and family members) both positively and negatively affect the circumcision decision-making of women. Acceptance occurs when the extended family (in-laws) agree to the procedure (Amuri et al., 2016). Social norms shaping gender roles were evident in a Zambian study in which women required permission from their husbands to pursue early infant male circumcision (EIMC) (Waters et al., 2012).

In South Africa, various cultural groups practice traditional male circumcision. Traditional male circumcision occurs at puberty as a rite of passage from boyhood to manhood (Ntozini & Abdullahi, 2018). Within the traditional practice of male circumcision, masculinity is central to the teaching at initiation schools (Douglas & Maluleke, 2018; Siweya et al., 2018). These teachings include culturally grounded expectations for men’s behaviours, roles, and relationships, linked to male dominance, power over women, and that only males should decide on circumcision (Sedibe, 2019).

Social norms influence how women make decisions about ICMC. However, little is known about the social perceptions of women regarding ICMC. According to Dempsey et al. (2018) social norms are unwritten individual expectations or rules about acceptable (and unacceptable) interactions. Against this background, this article analyses women’s perceptions of ICMC and proposes a perceptions framework for ICMC decision-making to inform ICMC policy.

## Theoretical Framework

This study uses the Social Norms Theory developed by Perkins and Berkowitz (1986) who addressed the problem of student alcohol consumption. The Social Norms Theory provides an understanding of the environment and interactive influences that modify individual behaviour (Dempsey et al., 2018). According to Social Norms Theory, behaviour depends on individual beliefs and perceptions of other social-group members’ practices and behaviour (Scholly et al., 2005). Social norms are “rules and standards that members of a group understand, and that guide or constrain social behaviours without the force of law” (Cialdini & Trost, 1998:152) and they link to perceived social pressure to participate or not participate in a behaviour (Ajzen, 2019). Social norms influence individual behaviours because they depend on people’s personal beliefs and decisions in specific situations.

The Social Norms Theory assumes that individual behaviour is influenced by perceptions of what others accept and expect. Generally, people think others engage in more negative behaviours than they do. Correcting these misperceptions will strengthen individuals’ feeling that their desire to resist negative behaviours is normal. In turn, this will increase a sense of social support for positive behaviours (Hahn-Smith & Springer, 2005)**.**

In public-health research, the Social Norms Theory has been applied in the context of HIV prevention. For example, one study of sexual behaviour practices and perceptions found that students held misperceptions about their peers’ levels of sexual activity and condom use (Scholly et al., 2005). Specifically, students significantly overestimated the number of partners, the level of sexual activity, the frequency of unintended pregnancies and the incidence of sexually transmitted infections among their peers, while underestimating the level of condom use. The Social Norms Theory can help with understanding the perceptions and socially constructed meanings attached to circumcision, which influence the acceptance and rejection of ICMC (Fleming et al., 2016). Therefore, policymakers can integrate Social Norms Theory perspectives into designing interventions to provide information that challenges social norms and misperceptions about ICMC. Using the Social Norms Theory as a theoretical framework, this study uses women’s voices to inform the development of a perceptions framework for ICMC decision-making.

The Social Norms Theory has been criticised for promoting positive health behaviours and not acknowledging the complexities of human behaviour that result from social norms evolving (Rachlinski, 2000). Furthermore, Berkowitz (2003) advised that, when employing the Social Norms Theory, researchers should ensure robust data-collection techniques to ensure reliable data and the development of strong normative education, information and communication messages. A failure to do so can lead to reinforcing misperceptions and misrepresentation. Davis et al. (2015) argued that, although the Social Norms Theory is extensive, it fails to illustrate implementation processes.

## Methods

### Setting

The study was undertaken in Diepkloof and Diepsloot, Johannesburg, South Africa. The sites were selected because the City of Johannesburg is a central metropolitan municipality with a sizeable HIV-positive population and one of the priority districts funded by the US President’s Emergency Plan for AIDS Relief (PEPFAR) (Van Schalkwyk et al., 2021). PEPFAR supports the voluntary medical male circumcision programme at the local public-health clinics in the two townships.

Located in the north of Johannesburg, 28.6% of households in Diepsloot are female-headed and in the township of Soweto, where Diepkloof is located, the percentage of female-headed homes is 40.3% (Statistics South Africa, 2013). The South African national HIV prevalence, incidence, and behaviour survey (Simbayi et al., 2019) reported that the Gauteng province has an HIV prevalence of 17.6% among 15–49-year-olds. However, the prevalence among pregnant women is 32.2% (Moyo et al., 2020; Simbayi et al., 2019).

The WHO (2009) indicated that the median age for undergoing traditional male circumcision in the Gauteng province is 17. Furthermore, 10% of the males aged 14–24 years and 22% aged 19–29 years were medically circumcised, and 58–65% of males underwent traditional male circumcision (Lagarde et al., 2003; Rain-Taljaard et al., 2004). In a male circumcision study conducted in Gauteng, participants self-reported a three-week healing period for traditional male circumcision (Lagarde et al., 2003).

The Human Sciences Research Ethics Committee of the Faculty of Health Sciences, University of the Free State (UFS HSD 2018/1443) provided ethical clearance. Informed consent was obtained from all participants before their inclusion in the study and the authors were respectful of the participants’ dignity and rights. The study followed the ethical standards of the 1964 Declaration of Helsinki and its later amendments. The national and Gauteng departments of health, South Africa, authorised the study. Furthermore, the study excluded information that could identify participants in the analysis and followed all standard ethics procedures of written informed consent, voluntary participation, and confidentiality. Pseudonyms were used to identify the participants in the study.

### Research Design

A qualitative descriptive research design guided this study. This design was appropriate because it generates an understanding of people’s perspectives by obtaining the meanings people attach to them (Caelli et al., 2003). A qualitative descriptive research design enables the collection of rich, detailed descriptions of a phenomenon for which limited information is available (Bradshaw et al., 2017). This study followed the consolidated criteria for reporting qualitative research (COREQ) (Tong et al., 2007).

### Sample

Purposive and snowball sampling were used to select South African Black women who had chosen not to circumcise their sons as infants or children. To recruit participants, a pamphlet was distributed in Diepkloof and Diepsloot. Women who expressed interest contacted the first author, who arranged the interviews. Snowball sampling helped to identify additional Black women. This process continued until we reached data saturation, and 25 participants were recruited. The snowball sampling method depended on referrals from the first participant to other participants who met the selection criteria. This sampling technique is useful when studying specific populations and discussing sensitive issues (Magnani et al., 2005). Data saturation was attained when no additional data were coming to light (Trotter, 2003).

The study sample consisted of 25 Black South African women. The participants were between 21 and 52 years old. All the participants had sons whom they chose not to be circumcised medically. Fourteen women obtained their high school qualification, nine completed vocational training, and two had a tertiary qualification. Seventeen women were unemployed and eight were employed part-time in the services sector. The sample represented Zulu, Venda, Tsonga, Sotho, and Xhosa ethnic groups, as self-identified by the participants. The ethnicities in the study were the dominant ethnic groups in the geographical research area. Furthermore, in the South African context, these Black population groups form part of the major ethnic groups in the country. The majority of the women were in relationships, and some were married. Most women lived with their intimate partners, while some lived with their parents. Participants were asked to report their ethnic group, religion, employment, relationship status, education level and their son’s circumcision status.

### Data Collection

Semi-structured interviews were conducted with Black women at the two study sites. One strength of a semi-structured interview is that it provides answers to the research question and a conversational mode of interaction, enabling participants to express their views spontaneously (Damons, 2008). The Social Norms Theory and the research questions related to ICMC information, traditional male circumcision and public health experiences, which guided the interviews. All the participants who volunteered to partake in the study agreed that the discussions in English could be audio recorded. The researcher recorded field notes in a separate notebook as observational insights for the study. Informed consent was obtained by administering a written consent form and an information leaflet containing an overview of the study. The participants signed the consent form, and all the interviews were conducted at a venue identified by the participants. Interviews between the participants and the lead interviewer took 40–60 min. Two follow-up discussions were arranged to answer questions that required further explanation.

The researchers used member-checking, by which transcripts were returned to participants to check for accuracy and correspondence with their experiences. The authors were trained in qualitative data collection to perform the in-person interviews. The lead author, who conducted the interviews, established a relationship with the participants by being the lead contact throughout the study and showing interest, respect and appreciation for the participants' time and effort to participate. The participants were aware that the lead author is a Black mother of a young child under five years old and a public-health specialist specialising in medical male circumcision and HIV-prevention programmes and policies.

### Data Analysis

**Fig. 1:**
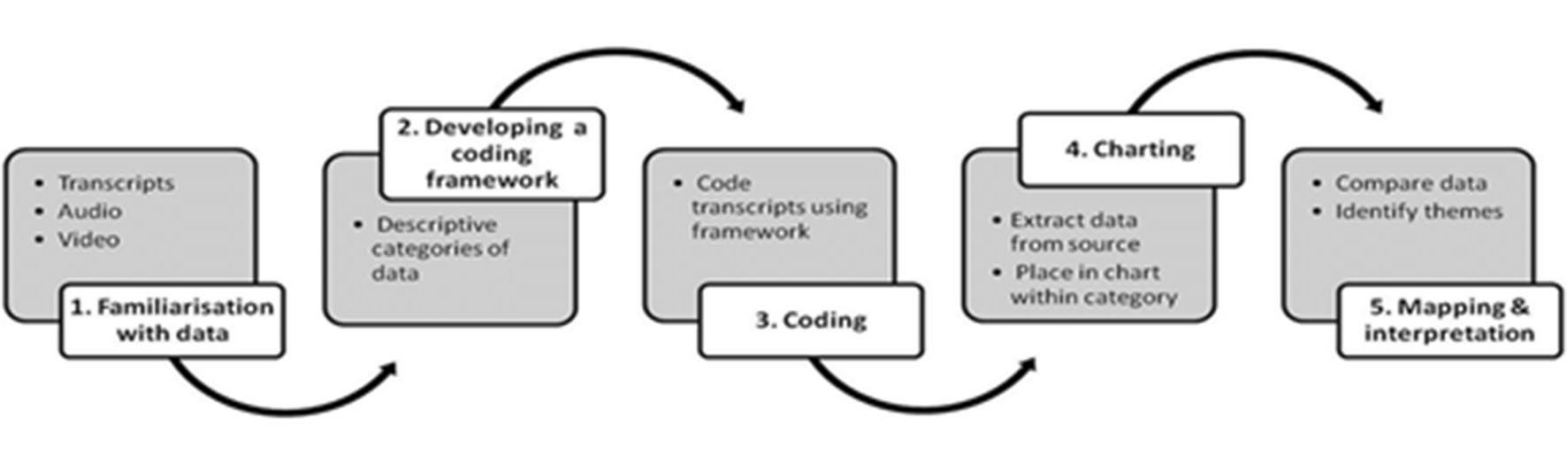
Five-step process in framework analysis (Source: Ritchie and Spencer 1994)

A framework analysis approach was used to analyse the data. Framework analysis is appropriate because it involves a comprehensive and systematic data-analysis process based on the initial responses of participants (Satyanarayana & Srivastava, 2010). Additionally, framework analysis is a flexible tool that allows for a comprehensive and detailed analysis of themes (Richie & Spencer, 1994). The Social Norms Theory analysis of the data focused on personal and social factors influencing the perceptions of ICMC. First, we familiarised ourselves with the data. Second, a coding framework was developed and, third, we extracted and synthesised the data to develop the final coding framework, a process termed charting. Finally, mapping and interpretation were made in which all themes were represented and the relationships between the themes were analysed (Fig. 1). The lead researcher transcribed the audio-recorded interviews verbatim. Transcriptions were then exported to Nvivo 12 (QSR International, Melbourne, Australia), a qualitative data management software package. The lead author used framework analysis to analyse the data. The principal investigator (EP) performed data coding and analysis with additional verifications by the co-authors (LM and ME).

## Findings

We identified the following themes derived from the data and direct verbal feedback from participants: perceptions of medical mistrust, cultural perceptions regarding traditional male circumcision, and inaccurate information leading to myths and misconceptions.

### Mistrust of the Public Healthcare System

The participants believed that the public healthcare system was unreliable, and their healthcare experiences informed their decision-making. Perceptions of mistrust resulted from women hearing, watching, and sharing historical problems. Zama (aged 42) remarked, “*I heard from my grandmother about how they were treated as women and my mother saying nothing has changed. So, I’m thinking, how can you listen and trust what they [government] say?”* This quote shows the historical roots related to the poor treatment of Black women in healthcare settings. Based on this experience, women expect their grandmother's experience to repeat itself. Therefore, women in the study did not accept ICMC because they did not trust the advice of public healthcare officials.

Second, observing problems in the public healthcare system created mistrust. Thandi (aged 27) said: *“I saw women giving birth in the lines and toilets because nurses are rude. So, imagine coming with an infant with problems from circumcision? I’m scared.”* This quote implies that some women perceived that public healthcare professionals do not care about patients, which sparked a sense of indignation among the participants. Being scared to be a victim of poor healthcare services inhibits women from deciding about ICMC.

Third, the women feared that help might be unavailable when requested. Vicarious experiences of another woman’s negative experience with her son’s circumcision were evident. Pinkie (aged 32) described her experience: *“My neighbour’s son was circumcised when he was young. It was a nightmare, she said. He had an infection, he was sick, and it took a long time to heal.”* The exposure to negative circumcision stories of others that produced undesirable surgical results could lead to higher levels of medical mistrust in the circumcision procedure.

The discussions with the women triggered intense emotional reactions and feelings of frustration, anger, and fear, when two women shared their personal experiences about the healthcare system. They perceived free healthcare services as inferior because of the post-operative complications related to their sons’ medical circumcision procedures.

### Cultural Perceptions

The cultural practice of traditional male circumcision may influence women’s perceptions of ICMC. Many participants said that ICMC goes against their beliefs and values of practising traditional male circumcision. Consequently, they did not accept ICMC. Santo (aged 37) voiced her strong cultural beliefs: “*We cannot just wake up in the morning and say that we will go for the child circumcision; what about our culture? We send boys to the mountain for circumcision in our culture [Tsonga].”* This quote shows that the cultural practice of traditional male circumcision forms the foundation of some women’s male circumcision views. The need for cultural expression resonates with Dineo (aged 31), who indicated that her son will be going to initiation school: “*I still believe in the old ways of doing things the right way, culturally and I don’t see anything wrong with that – my son will be going to the mountain soon.”* These quotes show that traditional male circumcision perpetuates traditional knowledge transfer within their cultures. Many participants subscribed to conventional structures and authorities, which reject ICMC.

The women who declined ICMC shared that ICMC altered many social values and traditions and reiterated their acceptance of and commitment to traditional male circumcision*.* Palesa (aged 52) mentioned that her sons underwent traditional male circumcision: *“Let me tell you straight, our people must be careful and not just follow new things [ICMC]; they try to cut culture out of everything; we must not be forced to do things that other people do.*” The quotes reiterate the women’s suspicion of ICMC and reinforce the fear that medical male circumcision practices are advanced at the expense of traditional male circumcision because African beliefs and practices are often viewed as problematic. Furthermore, the fear of cultural erosion and potential conflict between traditional practices and Western healthcare interventions is evident. Women perceive ICMC negatively because of the cultural decline in the value of traditional male circumcision. The foundations of traditional male circumcision teachings and cultural norms are passed down between generations to reaffirm women’s perceptions.

Two women were concerned that accepting ICMC could lead to social sanctions in the community if they decided to pursue ICMC. Such sanctions would ordinarily be exercised and lead to social rejection and, ultimately, isolation. Fulu (aged 31) said: “*When you circumcise in the hospital, they [other boys in the community] make fun of them and don’t treat them well. They treat them horribly.”* Sara (aged 23) supported this by saying: *“They call them ‘a man-woman’; it means a man by name and because he is not from circumcision school, he is called a woman. I don’t want my son to be treated like that”*.

The Social Norms Theory predicts that interventions designed to correct misconceptions will benefit some individuals. They may participate less in potentially harmful behaviour (traditional male circumcision) or be encouraged to engage in protective, healthy HIV-prevention behaviours such as ICMC. Social norms develop through women’s perceptions that they are not living up to the socially accepted practices of traditional male circumcision. If their sons do not undergo traditional male circumcision, they could face social interaction difficulties, judgement, and ill-treatment. The perceived negative labels constructed and assigned to boys and men who have not undertaken traditional male circumcision negatively shape women’s ICMC perceptions and decisions about ICMC.

### Inaccurate Information About Infant and Child Male Circumcision

Information is a prerequisite for healthcare decision-making processes. Some women said their unfavourable stance emanated from their lack of information about ICMC. The women who declined ICMC received limited information about ICMC from healthcare providers that fuelled their misconceptions about the procedure. These women often regarded their friends as a major source of information. Fernández (2013) found that women may not always receive sufficient information. Decisions may depend on the long-standing generational norms that may impact the availability of ICMC to women and the expectation of women to participate in ICMC decision-making.

Significantly evident from women’s experiences is that societal rules often govern the availability of ICMC information. Hlami (aged 36) considered it socially unacceptable for women to participate in family discussions about male circumcision: *“I do not know much about circumcision of infants because it’s not something that is talked about with women in our family. Only the men will talk if it comes up.”* Echoing her sentiments, Dineo (aged 30) described the exclusion and invisibility of women and fathers “managing” the information that is shared: “*We don’t feature when it comes to the circumcision of our sons, so you end up not knowing much; their fathers are in charge; they make decisions, so you only get information here and there.”* These quotes magnify gender-related social norms and power relations in spaces where circumcision decisions are made. Cultural beliefs related to traditional male circumcision dictate that it is taboo for women to participate in discussions about male circumcision.

The women who declined ICMC mentioned that their ICMC decision-making practices were informed by their perceptions of other women’s ICMC choices and expectations through connections with members of their social networks. Martha (aged 26) explained that her friends played a significant role in her decision to reject ICMC: *“Our friends have a lot of power. If they are against it [ICMC], you will also not do it [ICMC].”* Although the women regarded friends as a source of ICMC information, they also complained that their friends expected them to emulate their ICMC decisions. Anele (aged 22) said: *“It’s small, but it’s there. When you ask [friends] and they say they don’t support ICMC, it’s going to be awkward for you if you change your mind later.”* These women considered ICMC information from proximal reference groups such as friends signifying subtle, in-direct pressure to conform with the group decision (ICMC rejection). Social proofing is evident due to the normative social influence of friends, as women often conform (by rejecting ICMC) to gain acceptance by their social networks. Therefore, this social acceptance demonstrates that peers can profoundly influence women’s perceptions of norms related to ICMC. Furthermore, the women who declined ICMC described the information they gathered from social networks. For example, Nandi (aged 34) indicated that she received subjective, negative, and superficial detail on ICMC. She said: *“They [friends and family] tell you that you won’t sleep; they [children] cry a lot and there’s a lot of pain; so obviously, I’m not going that route.”* Even though the women receive inaccurate, misleading information from friends and family, they regard it as reliable because it rejects ICMC. Furthermore, the inaccurate information could also be consistent with pre-existing negative perceptions of the social network.

## Discussion

The negative perceptions of Black women towards ICMC circumcision presented in the current study resulted from medical mistrust, cultural practices related to traditional male circumcision and a lack of information about the procedure. These perceptions could represent significant barriers to seeking ICMC services for HIV prevention.

Multiple generations of women shared their perceptions of the public healthcare system. Intergenerational mistrust of the public healthcare system eroded trust. Many problems in the South African healthcare system result from the apartheid period (1948–1993). The healthcare system was highly fragmented, with discriminatory effects between different racial groups (Baker, 2010). Even though the democratic South African government is introducing significant amendments to healthcare policy and legislation, compliance, safety, efficiency, and quality concerns remain challenging (Mogashoa & Pelser, 2014; Moyakhe, 2014).

The South African history of racist policies, the legacy of abuse and government misinformation regarding the HIV epidemic has fuelled mistrust in healthcare services (Nattrass, 2013; Tun et al., 2012). (Sacks et al., 2021) noted that healthcare decision-making in Black communities depends on intergenerational experiences, mistrust, marginalisation and abuse. South Africa has experienced a high disease burden and an increased patient load, which negatively impacts the availability of healthcare services (South Africa & Department of Health, 2017).

Maphumulo and Bhengu (2019) stated that the South African healthcare system needed repair. Despite efforts of the government to improve the quality of healthcare services, the standards of care and patient expectations remained largely unmet (Visser et al., 2012). The public has lost trust in the healthcare system, leading to a decline in the use of public healthcare services by a population dependent on these services (Malakoane et al., 2020).

Furthermore, despite the limited political intention to improve healthcare services for women in the public sector, the participants have not received adequate information, counselling, and options about medical procedures and testing regarding maternal and child health (Campbell & Nair, 2014). Mavhu et al. (2014) highlighted the need to address social norms to improve the acceptability of EIMC because of mistrust based on experiences from medical male circumcision programmes. A similar medical male circumcision study in Eswatini highlighted concerns about the mistrust of HIV programmes (Adams & Moyer, 2015).

In addition to the perceptions of poor service quality of the public healthcare system, personal and anecdotal experiences of adverse events experienced by children negatively influenced women’s decision-making. This finding confirms a neonatal male circumcision study in Zambia that highlighted that the most cited reason among women who did not accept neonatal circumcision was the lack of trust in the medical personnel (Waters et al., 2013). Consistent with prior research on the acceptability of neonatal male circumcision in Zambia, the women in this study highlighted their fears related to the negative outcomes of the procedure (Waters et al., 2013).

A global review of neonatal and child male circumcision highlighted that the rate of adverse events increases when healthcare providers lack experience, adequate training and supplies (Weiss et al., 2010). According to Rech et al. (2014) rapid upscaling of the male circumcision programme in South Africa has decreased the quality of circumcision services regarding the aspects of adverse events, infection control, post-operative counselling, and external supervision. If South Africa should consider ICMC as an HIV-prevention strategy, the perception of medical malpractice and service quality requires attention.

Strong cultural beliefs related to traditional male circumcision profoundly influenced the women’s perceptions regarding ICMC. Traditional male circumcision represents a celebrated cultural practice that symbolises the transition from a boy to a man by constructing masculine identities (Gwata, 2009; Mavundla et al., 2009). Traditional male circumcision practices operate within social and cultural settings as cultural expression and identity. The women in this study believed that ICMC interfered with their cultural beliefs and practices and were concerned that their culture was threatened. This finding is consistent with two independent studies of neonatal male circumcision conducted in Zambia and eSwatini. Women indicated that ICMC was not part of their cultural and traditional beliefs and rejected the practice (Jarrett et al., 2014; Nyoni, 2015). In a South African study, women indicated that ICMC excluded important traditional practices embedded in their cultural beliefs (Phili & Karim, 2015). Black women’s male circumcision objections were influenced by their perceptions that male circumcision programmes could lead to cultural erosion and the destruction of customary practices (Spyrelis et al., 2013).

Traditional male circumcision practice contains notions of masculinity linked to affording rights, privileges and social benefits attached to access to resources and inclusion in community and family rituals (Ntombana, 2011). The social construct of manhood and masculinity have been deemed challenging as medically circumcised men are viewed as inferior to traditionally circumcised men (Mavundla et al., 2009, 2010; Peltzer & Kanta, 2009). Our study confirms this reality. In one acceptability and feasibility study conducted in Malawi, women identified cultural considerations as a barrier to accepting the procedure due to traditional beliefs related to traditional male circumcision that dictate that a woman should not see her son’s circumcised penis (Chilimampunga et al., 2017). Our findings show that women resisted ICMC because it would erode African cultural values and traditions. According to Kalichman (2010) argued that in high-HIV-prevalence settings, African cultures reject neonatal male circumcision because in traditional male circumcision practices, strong perceptions are held about masculinity and maturity. This demonstrates scepticism about ICMC in Africa because of the dominating biomedical approaches followed in Western cultures (Niang & Boiro, 2007).

Black women who declined ICMC feel disempowered in the ICMC decision-making process due to limited information and the fathers’ leading role in the decision-making process. As Chilimampunga et al. (2017) pointed out, women’s perceptions of EIMC were influenced by fathers making circumcision decisions. The dominance of fathers constrained the participation of women in the decision-making process. In a Zimbabwean acceptability study of EIMC, participants considered it taboo for “outsiders", including women and children, to access information about medical circumcision (Mavhu et al., 2012). Furthermore, the study showed that women also conform by not challenging ICMC decisions because they are from a different clan from their husbands and are unfamiliar with the clan’s circumcision practices (Mavhu et al., 2012). Therefore, women conform to social expectations of silence and reinforce male dominance in decision-making. Thus, restrictive gendered social norms cement the collective belief of the dominant role men should play in the decision-making process, thereby reinforcing women’s exclusion, ostracisation and marginalisation in the decision-making process. Furthermore, social constructions of masculinities characterised by male dominance, strength, power, giving permission and leading women are the main feature in decision-making (Shefer et al., 2007).

The relationship between the social network and women’s negative perceptions was significant for women in this study. The social context shapes perceptions at the interpersonal level. Women use a network of social relationships (friends and family) to access information about ICMC, creating subtle social pressure among women to conform. Conforming to what other members of the social reference group (friends and family) expect maintains social norms because of the fear of rejection or sanction (Cislaghi & Shakya, 2018). Social proofing is evident due to the normative social influence of friends, as women often conform by rejecting ICMC, so as to gain acceptance by their social networks. Therefore, this social acceptance demonstrates that peers can profoundly influence women’s perceptions of norms related to ICMC. The concept of social proofing is considered a social, psychological phenomenon whereby individuals copy the actions of others to inform decision-making (Cialdini, 2001). Therefore, social proofing influences ICMC decisions because Black women follow the steps and decisions of their friends and conform because of a fear of being rejected or disliked, promoting perceptions of ICMC rejection.

Women in both a South African and a Zimbabwean study indicated that friends play an important role in women deciding to accept or reject EIMC (Mavhu et al., 2012; Spyrelis et al., 2013). Despite using social networks as sources of information, they often do not have accurate medical information about neonatal male circumcision (Pruenglampoo, 2015). Therefore, recognising the powerful role of social networks underscores the need to provide real, targeted, credible risk–benefit ICMC information to parents, which is vital to support decision-making (Waters et al., 2012).

**Fig. 2:**
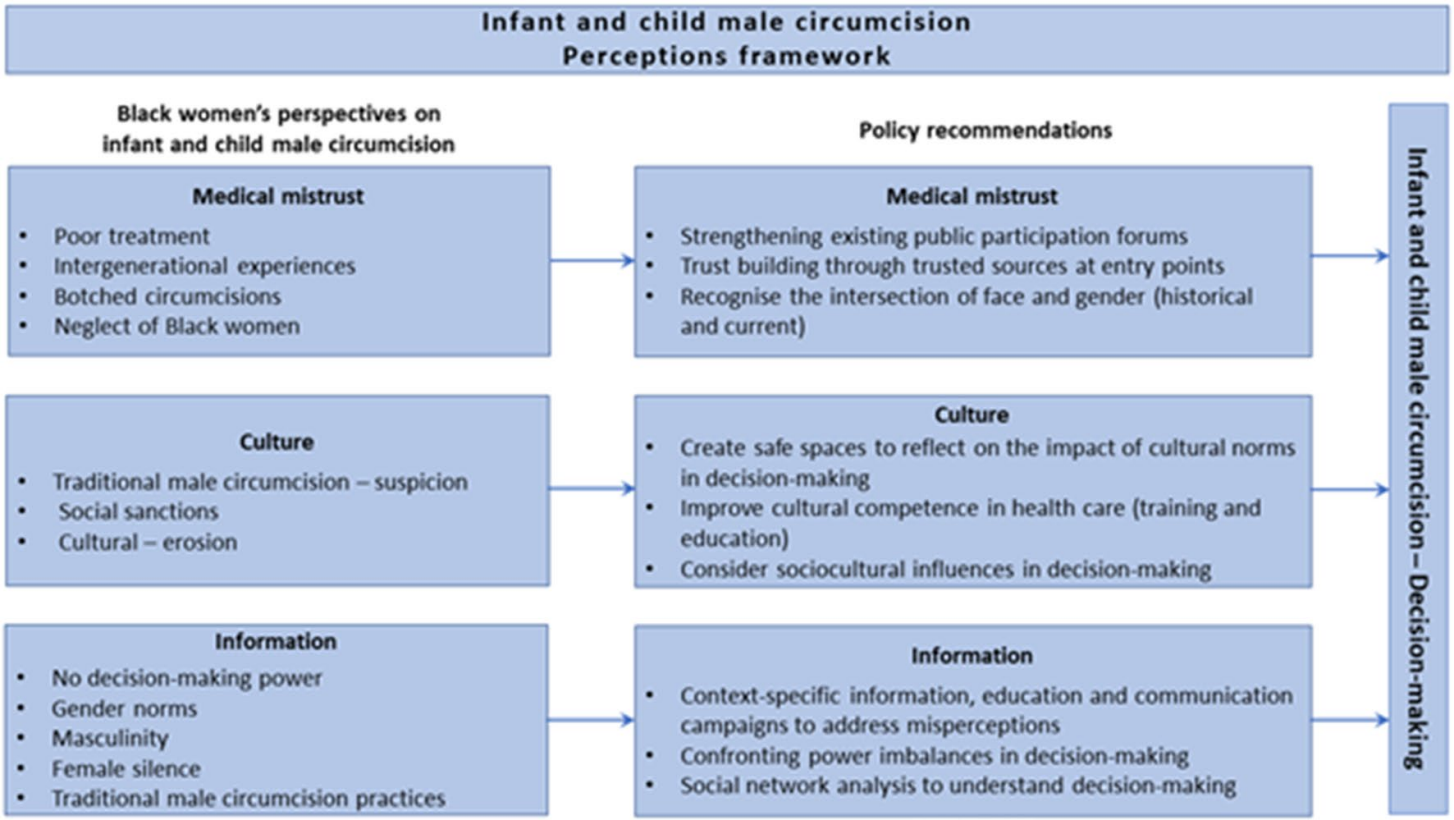
Infant and child male circumcision perceptions framework (Palmer et al., 2022)

Based on the findings, Fig. 2 depicts the contribution of the study as captured in the recommendations below:

## Recommendations

We make the following recommendations to inform medical male circumcision policies and programmes:

- **Understand stakeholders and context:** To implement an ICMC policy, a social network analysis can be a tool to map and measure the relationships between stakeholders involved in ICMC decision-making. Such analysis could identify the main network actors and describe the interactions involved to provide the context for understanding the social norms that influence the ICMC decision-making process.
- **Revise gender applications and approaches to ICMC decision-making**: Advancing gendered approaches in male circumcision policy frameworks sets the scene for a contextual understanding of the role of women in ICMC decision-making and widens the scope for social inclusivity. Therefore, emphasising gender dimensions related to power imbalances and the equal participation of women in ICMC decision-making processes is needed. The focus should be on addressing the domination of men in decision-making and women's increased representation and participation in decision-making.
- **Consider women’s access to healthcare service needs:** Embed and recognise the role of social norms in ICMC decision-making within male circumcision policy domains. Additionally, the needs of women should be recognised in the delivery of healthcare services, with a focus on cultural sensitivity in the context of a diversity of values and beliefs, recognising the organisational, clinical, structural, and sociocultural barriers women face in health and healthcare.
- **Promote trust in public healthcare systems:** Policy frameworks acknowledging the complexities of trust in the South African public healthcare system can go a long way in addressing medical mistrust. Initiatives that address social norms and maintain trust in the context of patient-centred care for women are important. Furthermore, interventions should be responsive and respectful to inform male circumcision policies to improve women's and their sons’ healthcare and health outcomes.
- **Mobilise communities:** Utilising community leaders as entry points for community mobilisation and participation in interventions related to social norms. Promoting dialogue through reflection about ICMC decision-making barriers that women who declined ICMC face, linked with concrete actions, can facilitate addressing social norms related to ICMC decisions.
- **Encourage continuous dialogue**: Cultivating safe places for reflection through constructive dialogue to understand the social norms that negatively impact ICMC decision-making, specifically related to traditional male circumcision practices, medical mistrust, and misinformation. Moreover, a focus on building environments in which women can be emotionally safe to share their experiences and concerns and ask questions is needed.

### Limitations

This study was limited to Black women who did not opt for ICMC in Diepsloot and Diepkloof, Gauteng, South Africa. Therefore, the findings are contextual and may not necessarily apply to other communities in Gauteng and the broader South African society. Nonetheless, the results offer insights into Black women’s perceptions of ICMC that could inform ICMC policies and programmes. Even though women who declined ICMC were the primary focus of the interviews, additional information could come from fathers and other influential family members. The quality of the study findings may have been affected because all the interviews were in English, and some participants may have faced language barriers.

## Conclusion

Social experiences and historical and cultural contexts influence how women make ICMC decisions, which influence the effectiveness of ICMC as an HIV-prevention strategy. Social norms distort Black women’s perception of ICMC negatively. This study makes several contributions to the literature on ICMC decision-making. First, the study demonstrates that Black women who declined ICMC in this study experience ICMC negatively because they experience social pressure from their social network of friends to conform to the negative views of ICMC. Furthermore, men lead ICMC decision-making within the family context, reinforcing historically unequal gender relations and limiting equal participation. Second, the study highlights the need for evidence-based and theoretically grounded policies to guide interventions that effectively influence social norms. Addressing the negative perceptions of ICMC directly by understanding women’s ICMC beliefs and unique experiences may be valuable to understanding complex decision-making processes. Third, this qualitative descriptive study provides several empirical findings for medical male circumcision policy and programmes within the complex sociocultural context of traditional male circumcision practices. Policies and programmes addressing social norms in disfavouring some of the harmful practices of traditional male circumcision as it relates to Black women who declined ICMC could be implemented and evaluated, recognising that this remains a topic of contestation. Fourth, the study demonstrates the importance of considering gendered perspectives in the medical male circumcision discourse and the need for an inclusive knowledge base that amplifies Black women’s voices in HIV prevention.

## Data Availability

All original data is securely stored and available, as is information regarding data analysis.

## Abbreviations

ICMC: Infant and child male circumcision
HIV: Human immunodeficiency virus
PEPFAR: US President’s Emergency Plan for AIDS Relief
UNAIDS: Joint United Nations Programme on HIV/AIDS
WHO: World Health Organisation
